# Notochord Cells in Intervertebral Disc Development and Degeneration

**DOI:** 10.3390/jdb4010003

**Published:** 2016-01-21

**Authors:** Matthew R. McCann, Cheryle A. Séguin

**Affiliations:** 1 Department of Physiology and Pharmacology, Schulich School of Medicine & Dentistry, Western University, London, ON N6A 5C1, Canada; mmccann4@uwo.ca; 2 Bone and Joint Institute, Western University, London, ON N6A 5C1, Canada

**Keywords:** animal models, disc degeneration, intervertebral disc, notochord, nucleus pulposus

## Abstract

The intervertebral disc is a complex structure responsible for flexibility, multi-axial motion, and load transmission throughout the spine. Importantly, degeneration of the intervertebral disc is thought to be an initiating factor for back pain. Due to a lack of understanding of the pathways that govern disc degeneration, there are currently no disease-modifying treatments to delay or prevent degenerative disc disease. This review presents an overview of our current understanding of the developmental processes that regulate intervertebral disc formation, with particular emphasis on the role of the notochord and notochord-derived cells in disc homeostasis and how their loss can result in degeneration. We then describe the role of small animal models in understanding the development of the disc and their use to interrogate disc degeneration and associated pathologies. Finally, we highlight essential development pathways that are associated with disc degeneration and/or implicated in the reparative response of the tissue that might serve as targets for future therapeutic approaches.

## 1. Introduction

“Know from whence you came. If you know whence you came, there are absolutely no limitations to where you can go.”

—James Baldwin

In trying to understand the cellular and molecular basis of tissue degeneration, it is important to consider the dynamic interplay involving cell-cell communication, gene regulation, and growth factor and cytokine secretion required to form a healthy, functional tissue during normal development. Once we have identified pathways that serve to regulate tissue formation, we can start to investigate alterations in these signals during normal aging, disease, and tissue repair.

Low back pain has become one of the most common causes of disability, affecting overall well-being and work performance, with recent reports indicating a lifetime prevalence as high as 85% in industrialized countries [1]. In fact, according to the recent *Global Burden of Disease* study, low back pain is the most common musculoskeletal disorder and the leading cause of years lived with disability in all developed countries [2]. The prevalence of chronic low back pain has increased remarkably over the last decade for all ages and genders; increases have been reported to be as high as 162% between 1992 and 2006 in middle-aged individuals in the US [3]. Alarmingly, there are no disease-modifying therapies for low back pain and current treatment options do not adequately provide improved outcomes [4]. While several risk factors for back pain have been identified such as, obesity [5], psychological factors [6], age and sex [7], and genetic variants [8], the molecular cause(s) of back pain remain elusive.

Although the etiology of low back pain is unknown, it is often associated with degeneration of the intervertebral disc (IVD). The current lack of disease-modifying therapeutics for disc degeneration is linked to a poor understanding of the biological mechanisms that regulate IVD development, health and degeneration. This review provides an overview of IVD biology, with a specific emphasis on the development of the nucleus pulposus (NP) and how the embryonic notochord plays an essential role in adult IVD homeostasis. Furthermore, we explore the opportunities and challenges associated with the use of animal models to study the IVD.

## 2. Functions and Structure of the Intervertebral Disc

IVDs are complex connective tissue structures that serve to anchor adjacent vertebral bodies along the spinal column, essential for mechanical stabilization of the spine and load bearing during axial compression [9]. The cartilaginous joints formed by IVDs also provide flexibility and movement to the spinal column. The IVD consists of three distinct, yet interdependent specialized tissues: the central viscous NP, the outer fibrillar annulus fibrosus (AF), and the cartilage end-plates (CEP) that anchor the disc to the adjacent vertebral bones. The diversity in the structure of these tissues is related to the specific organization of their extracellular matrix (ECM), which is produced and maintained by distinct cell populations.

The NP is composed primarily of large aggregating proteoglycans held together loosely by an irregular network of type-II collagen and elastin fibers. The aggrecan monomers are highly sulphated by covalently bound glycosaminoglycans (GAGs) (chondroitin sulfate, keratin sulfate) and cross-linked to hyaluronic acid via cartilage link protein [10]. The GAGs confer a net negative charge to the aggrecan molecules, attracting large numbers of counter ions and therefore water molecules [11,12]. The influx of water to the NP is created by osmotic pressure [13], and the water molecules are retained in the matrix in part by the irregular meshwork of type II collagen [14]. The osmotic pressure provides the stiffness required for the NP to maintain height and turgor against compressive loads. With sustained loading, such as human daily activity, roughly 20%–25% of the water slowly diffuses out of the disc into the paravertebral muscles, resulting in a decrease in disc height [15,16]. Upon removal of the compressive load (*i.e.*, nightly rest), water is reabsorbed into the NP matrix, resulting in a re-expansion of the tissue and recovery of disc height [17,18,19].

The AF is a fibrocartilagenous structure formed by distinct lamellae composed of bundles of type I collagen fibers oriented at oblique angles, that alternate within each consecutive lamella to form an angle-ply structure (25 lamellae in the AF of a human disc) [20]. This arrangement provides mechanical support to resist the turgor pressure that is applied by the NP. The outer AF contains elastic fibers (elastin, elaunin and oxytalan proteins) that directly associate with the adjacent vertebra and extend into the bone as Sharpey’s fibers to anchor the IVD into bone [21]. The inner AF forms a transition zone between the fibrous outer AF and the gelatinous NP, containing higher levels of glycosaminoglycans and type-II collagen in the interlamellar matrix when compared to the outer AF [22].

The disc is anchored superiorly and inferiorly to the adjacent vertebral bodies through the CEPs, thin layers of hyaline cartilage that interface with the inner AF and NP. In humans, the adult NP is the largest avascular tissue in the body and therefore depends on nutrient, metabolic by-products and O_2_ gas exchange through the CEPs via passive diffusion from the highly vascularized vertebral bone [23]. The CEP is formed by chondrocytes similar to those found in articular cartilage, which secrete a type II collagen- and proteoglycan-rich extracellular matrix.

## 3. Intervertebral Disc Development

### 3.1. Early Embryo and Node Development

Much of our knowledge of mammalian notochord development is derived from experiments using mouse models due to their short gestation period, large litter sizes and ease of genetic manipulation. As such, the data presented below apply to the murine model and developmental stages, unless otherwise indicated.

**Figure 1:**
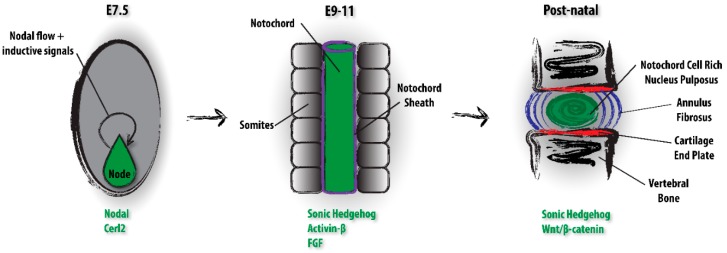
Schematic representation of key events in the transition from notochord to nucleus pulposus development in the murine model. Notochord cells within the mouse embryonic node at embryonic day 7.5 play a fundamental role in left/right patterning of the early embryo by secreting Nodal and Cerl-2. Notochord cells proliferate and migrate to form the embryonic notochord (surrounded by the notochord sheath) between embryonic day 9–11 where they provide instructive cues that pattern the neural tube and sclerotome through secretion of sonic hedgehog. Notochord cells then go on to form the mature nucleus pulposus (NP) in the post-natal intervertebral disc (IVD) (shown here in the sagittal plane), where they continue to secrete inductive factors, including sonic hedgehog and Wnt, which serve to regulate intervertebral disc homeostasis.

During gastrulation the primitive streak is formed along the longitudinal axis of the embryo, establishing longitudinal (anterior/posterior) and bilateral (left/right) symmetry. In the mouse, a cluster of cells located near the anterior end of the primitive streak ingress to form the node (Figure 1). The node is a transient, pit-shaped structure located at the distal tip the early embryo, responsible for establishing the left asymmetry of the body plan. Monocilia localized to the apical surface of node cells beat in a clockwise rotation (when viewed from the ventral side) to drive a leftward flow of extraembryonic fluid containing morphogens, such as Nodal and the Nodal antagonist Cerl-2, secreted by the columnar epithelial cells of ventral node [24,25,26,27]. This fluid flow promotes left side specification via two possible mechanisms: either through direct morphogen-induced differentiation on the left side of the embryo, or by inducing a mechanosensitive calcium signalling response to the nodal flow in the perinodal crown cells that promotes cell specification. While the underlying mechanisms remain to be established, the node and resultant nodal flow are necessary for asymmetric differentiation of the left axis of the embryo [28,29,30].

### 3.2. Notochord Development

In the mouse, the first stages of notochord development originate at the notochordal plate between E8.0–E8.5. This plate-like structure lays in the ventral midline of the embryo and is continuous with the dorsal gut endoderm. At E9.0, the notochord plate folds off the gut endoderm to form the anterior notochord, where it rests in a central position in the embryo, flanked by the dorsal ridge of the neural tube (the floor plate) and ventrally by the gut endoderm (the endoderm plate). Laterally, the notochord is flanked by the paraxial mesoderm, which will go on to form the somites and subsequently the AF and vertebrae.

Anatomically, the notochord is a continuous rod-like structure that forms the primitive anterior/posterior axis of the embryo. The notochord is able to resist compressive loads along the axis of the embryo yet is sufficiently flexible to resist buckling [31]. The unique structural and cellular features of the notochord achieve this rigidity. In mammals the notochord is surrounded by an acellular sheath composed of collagens, laminins and proteoglycans [32,33], while in zebrafish the sheath is made up of an epidermal cell layer; both serving to encapsulate the notochord [34]. In zebrafish, alterations in the composition of the notochord sheath, such as loss of *col15a1* or *emilin3*, result in specific phenotypes associated with notochord malformations [35,36,37,38]. Furthermore, disruptions in the structural integrity of the sheath result in loss of the embryonic axis and perturbations in spinal column development [33]. In mammals, the cell type responsible for secretion of the notochord sheath has yet to be established, with the extracellular matrix proteins produced by notochord cells, the surrounding mesenchyme cells or a combination thereof. Notochord cells also directly contribute to the mechanical properties of the notochord due to the presence of large intracellular vacuoles that increase cell size and occupy ~80% of the cell volume [39]. The presence of these vacuoles have been described in numerous species including amphibians [40], birds [41], mammals [42] and humans [43]. While conserved across species, their classification has been long debated. Recent work by Ellis *et al.* demonstrated that in zebrafish, notochord cell vacuoles are lysosome related organelles that require late endosomal trafficking and H^+^-ATPase-dependent acidification [44]. In contrast to typical lysosomes that have a low pH [45], V-ATPase in notochord vacuoles generates a proton rich gradient that allows water to flow into the vacuole through osmosis [46]. Work from our lab has shown that H^+^-ATPases are enriched in the mouse IVD [47], and others have indicated their importance in bovine [48] and canine [39] NP cells suggesting that the processes governing notochord vacuole formation maybe be conserved. The expansion of intracellular vacuoles contributes to longitudinal elongation of the embryonic axis; disruption of vacuolated cell differentiation or integrity in the zebrafish was shown to alter notochord and spine morphogenesis [44]. In mammals, the notochord remains in place until the development of the permanent axial skeleton (e.g., vertebrae, IVDs).

### 3.3. Notochord Signalling

During early development, the notochord is generally considered a signalling centre, secreting growth factors and morphogens responsible for patterning of surrounding tissues, including the neural tube [49], sclerotome [50], pancreas [51] and aorta [52]. The best characterized is its ability to direct neural tube neurogenesis during dorsal-ventral patterning. Mutations that disrupt notochord formation result in altered floor-plate induction [53]. Conversely, grafting experiments that placed an ectopic notochord adjacent to the neural tube established a secondary floor plate [49,54]. This process is thought to be mediated by notochord-derived SHH and chordin, as transplantation of microcarrier beads likewise resulted in the formation of a secondary floor plate [55].

The notochord also influences mesodermal muscle determination in the sclerotome; experiments have shown that mutations that prevent notochord development disrupt sclerotome formation [56]. In zebrafish, mutation of the *floating head* (*flh*) gene that disrupts notochord formation results in ectopic midline muscle development [57], suggesting that notochord-derived signals inhibit muscle development. This interaction appears to be mediated by pair-box transcription factors, specifically Pax1 induction and Pax-3 and Pax-7 repression [56,58]. Finally, the notochord is also known to contribute to endoderm patterning [59], where notochord-derived activin-βB and FGF2 have been shown to repress SHH in the endoderm thereby promoting pancreas development [60].

While the importance of the notochord in early embryonic development is well established, the postnatal role of notochord cells has been a source of ongoing controversy. Work from our lab and others have shown that in the mouse the notochord disappears in areas where the vertebral bodies form but expands within the perichordal disc, to form the central NP [61,62]. Interestingly, the mechanisms by which the notochord undergoes regression in the presumptive vertebrae are unknown [63,64]. The absence of detectable notochord cell apoptosis in this region or proliferation in the future NP [65] has led to the hypothesis that notochord cells either succumb to pressure from the developing bone mesenchyme and are forced into the future NP, or undergo directed migration to sites of the future NP [64]. Within the newly formed NP, notochord cells produce a glycosaminoglycan-rich extracellular matrix, which separates the original cell mass into a network of cell clusters.

### 3.4. Role of the Mesenchyme in Intervertebral Disc Development

Development of the axial skeleton is a multi-step process, beginning with notochord formation and requiring fine-tuned coordination and pre-patterning of individual skeletal segments. In the mouse, the paraxial mesoderm undergoes segmentation between E7.5–E11.5 to form the somites, which are metameric structures that exist transiently during vertebrate development [66]. The somites lie adjacent to the neural tube and the notochord. In response to bone morphogenetic protein (BMP) and Wnt signals from the ectoderm and Shh signaling from the notochord and neural tube floorplate, the dorsal aspect of the somites differentiate to form the dermomyotome, which gives rise to the dermis. At E12.5, the ventral portion of the somites differentiates to form the sclerotome that will give rise to the ribs, vertebral bodies and CEP and AF of the IVD [67]. The sclerotome cells migrate and aggregate around the notochord to form a continuous perichordal tube [68]. At E15.5, these mesenchymal cells acquire a metameric pattern of condensed and non-condensed segments along the anterior/posterior axis. This process is regulated by Notch and Wnt signalling to maintain somite polarity [69,70]. The notochord secretes Shh thereby regulating differentiation of the sclerotome in the formation of the axial skeleton. Notably, the vertebral column is not formed in mice lacking *Shh* [71]; however, targeted deletion of *Shh* in the floorplate does not alter IVD development, and deletion of *Shh* in both the floorplate and notochord results in the loss of IVD and vertebral body structures [72]. Lineage tracing using both the *Gdf5*-Cre [73] and *Tbx18*-Cre [74] demonstrated the mesenchymal origin of the AF. The condensed segments will later contribute to the mesenchyme-derived AF, whereas the less-condensed segments form the templates of the future vertebral bones.

### 3.5. Notochord Cell Contribution to the Nucleus Pulposus

Unlike the cells of the AF and CEP that remain relatively stable throughout life, the cells of the NP undergo drastic changes within the first decade of life in humans. In most vertebrates, including humans and mice, there is a progressive loss of large vacuolated notochord cells immediately after birth and the NP becomes populated by small cartilage-like nucleus pulposus cells [75,76]. Interestingly, the loss of the large vacuolated notochord cells from the NP is associated with the onset of disc degeneration, suggesting that these cells contribute to maintenance of the NP [75,77,78]. Despite this correlation, only recently has there been insight into the underlying biological mechanisms.

There have historically been two conflicting hypotheses regarding the origin of the cartilage-like cells of the NP. It was originally suggested that these cells were of mesenchymal origin, resulting from migration of cells to the NP from either the surrounding CEP [79] or the perichondrium at the periphery of the IVD [80]. During IVD formation, notochord cells were believed to direct mesenchymal cell migration, stimulate matrix synthesis, and then undergo apoptosis or necrosis [81,82]. More recent studies have suggested an alternate model in which notochord cells serve as NP progenitors and undergo terminal differentiation to give rise to the cartilage-like nucleus pulposus cells [77,83,84]. Our group and others conducted lineage-tracing studies in mice that established that during normal development and aging all cells of the NP are notochord-derived (from E15.5 until 21 months of age) [61,62]. To investigate the cellular composition of the NP during IVD degeneration, a mechanically-induced model of disc degeneration was used in mice where bone marrow-derived cells were labeled with GFP. These studies demonstrated an increased number of GFP^+^ BMSCs in the vertebral bone marrow, vascular canals of the CEP and the site of injury in the outer AF; however, BMSC-derived cells were not detected in the NP [85]. While other genetic approaches and *in vivo* models of disc degeneration or other spine-related pathologies should be used to examine the cellular composition of the IVD in various disease states, the data to date supports the notochord cell maturation model. In fact, recent publications in the field have suggested that mature cells within the NP should instead be referred to as “nucleus pulpocytes” to underscore their distinct developmental origin and function [86].

## 4. Intervertebral Disc Degeneration

**Figure 2:**
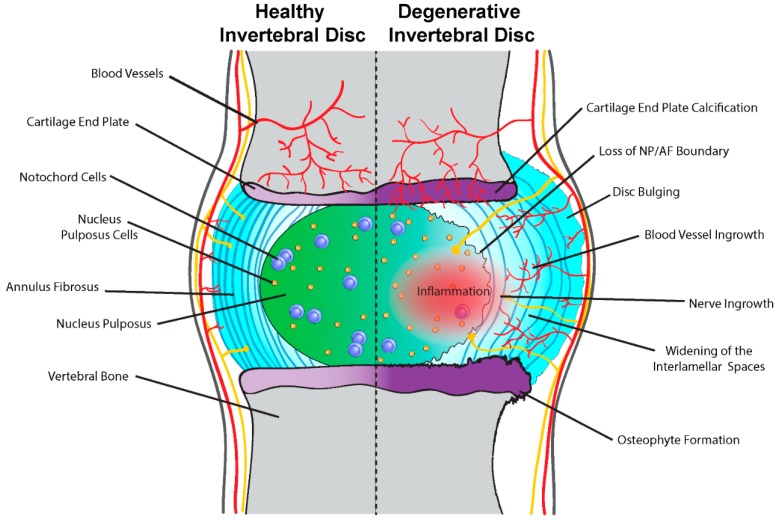
Schematic representation highlighting the hallmarks of human disc degeneration. Compared to healthy intervertebral disc, the degenerative disc has increased inflammation, blood vessel and neuronal ingrowth, loss of the boundary between the NP and annulus fibrosus (AF), and widening of the interlamellar space between collagen bundles in the AF that often results in disc bulging. In advanced degeneration the cartilage endplate calcifies and osteophytes form on the adjacent vertebral bones.

The most common cause of back pain is disc degeneration, the etiology of which is poorly understood. Consequently, there are no specific criteria to distinguish degeneration from the physiological processes of growth, aging or adaptive remodeling [18]. Disc degeneration has perhaps been best defined as a cell-mediated response to progressive structural failure [18]. This process is thought to initiate with changes to the cellular microenvironment within the disc and progress over decades, resulting in structural breakdown and functional deficiency [87,88]. Degeneration of the IVD is associated with increased extracellular matrix breakdown [89], abnormal (fibrotic) matrix synthesis [90], inflammation [91], and in-growth of nociceptive nerves and blood vessels into a typically aneural and avascular tissue [92] (Figure 2).

Initiation of the degenerative process is thought to originate specifically in the NP [93,94]. The deterioration of the major structural proteoglycan aggrecan [95,96], along with decreased production of other ECM components in the NP, lead to reduced hydration [97], loss of disc height, and an overall inability to absorb compressive load [98,99]. Compressive forces are therefore distributed to the surrounding AF, which results in altered mechanical properties and structural deterioration including radial and circumferential tears in the AF [100,101]. These tears often precede radial bulges or herniations of the NP substance into the adjacent spinal cord, resulting in pain [88,102]. Currently, there are symptomatic treatments for late stages of degenerative disc disease but no disease-modifying therapeutics [103].

### Notochord Cells and Intervertebral Disc Homeostasis

Within the postnatal IVD notochord cells are thought to maintain their role as important signaling effectors regulating IVD cell function. This function was first suggested by co-culture experiments that demonstrated the ability of notochord cells to increase proteoglycan synthesis in mature NP cells [78]. This effect was shown to be independent of direct cell contact, as notochord-condition media could recapitulate the anabolic effects of notochord cells on NP cells [104,105] as well as AF cells [106]. Notochord secreted factors, including the matricellular protein CCN2, directly regulate NP cell function by upregulating anabolic gene expression and downregulating catabolic gene expression [107,108,109]. Notochord secreted factors have also been shown to inhibit cell death and apoptosis of NP cells and protect NP cells from the degradative effects of cytokine exposure [110].

Since disc degeneration and LBP are associated with nerve and blood vessel in-growth to the IVD [92], studies have investigated the ability of notochord-secreted molecules to prevent axons and/or blood vessels from entering inappropriate territories. During mouse development, secretion of semaphorin 3E from the notochord and lateral plate mesoderm has been shown to regulate dorsal aorta formation [52]. In the IVD, notochord cells have been shown to inhibit angiogenesis by suppressing Vegf expression in endothelial cells [111]. With regards to neuronal patterning, studies of early chick development established that the notochord and dermamyotomes regulate axonal guidance [112]. These studies also identified semaphorin 3A as a notochord-derived axon repellent [113]. Interestingly, the outer AF of degenerative human discs shows reduced expression of semaphorin 3A compared to healthy IVDs, suggesting that IVD-associated nerve repellent pathways maybe be conserved across species [114]. In keeping with the correlation between the loss of notochord cells and neural in-growth to the IVD, expression of Nrp-1, another inhibitor of anxonal growth expressed in the NP, is decreased in degenerative human discs [114] and in aged rat NP [115].

The microenvironment of the NP may also contribute to repelling innervation. The ECM of the notochord differs from that of cartilage tissues in that it lacks keratin sulfate and has high levels of chondroitin sulfate [116]. In zebrafish, chondroitin sulfate has been shown to guide the development of the nervous system by inhibiting the formation of segmental motor neurons [117]. Co-culture studies demonstrated that chondroitin sulfate derived from the notochord or notochord sheath directly induced dorsal root ganglia cone collapse and increased axonal repulsion [118]. The role of notochord-derived chondroitin sulphate in restricting neural in-growth appears to be maintained in the adult IVD; recent studies report that soluble factors derived from porcine notochord cell conditioned media inhibit *in vitro* neurite outgrowth and that this affect was ablated with chondroitin sulfate digestion [119]. Interestingly, a genome-wide association study identified *CHST3* as a susceptibility gene for lumbar disc degeneration [120], and loss-of-function mutation in *CHST3* results in chondrodysplasia with major involvement of the spine [121]. *CHST3* is the gene encoding chondroitin 6-*O*-sulfotransferase, an enzyme required for proper chondroitin sulfate synthesis that is required for proper cartilage formation [122]. While chondroitin 6-*O*-sulfotransferase activity is required for water retention with the IVD [120], it may serve a dual purpose of also inhibiting neural in-growth [123,124]. Conversely, NP tissue is able to cause nerve tissue injury in the absence of mechanical compression [125], indicating that it is most likely chemical in nature.

## 5. Small Animal Models to Study Intervertebral Disc Development and Degeneration

### 5.1. Notochord Cells in Animal Models

Due to the difficulties associated with obtaining “healthy” intact human IVD tissue and cells or biological material from the early stages of disease onset, animal models have become invaluable to study the IVD biology [126]. Animal models have been incorporated into studies aimed at characterizing disc development, disease progression and to develop therapeutic interventions. In all mammals, development of the IVD involves common pathways associated with node formation, notochord elongation and aggregation of the mesenchyme around the central notochord (as described above). Although the overall process is similar, one important difference between species is the postnatal persistence of notochord cells in the NP—an important factor to consider when studying NP aging or degeneration. Unlike humans, species such as the rat, pig, cat, and rabbit, retain notochord cells throughout their life [16]. Large animals such as bovine and sheep better resemble humans in that the notochord cells present at birth rapidly decline in number early in life [126].

The use of canine models has revealed insights into the importance of notochord cells in IVD health [127,128]. While there is a large genetic variability in canine breeds, they can be classified as either chondrodystrophic or non-chondrodystrophic. Chondrodystrophia is altered development in the cartilage of the long bones, especially those involved the region of the epiphysial plates, resulting in arrested growth of the long bones [129]. Chondrodystrophic dogs (dachshund, bulldog, and beagle) have a higher prevalence and earlier age of onset of disc degeneration, while non-chondrodystrophic dogs are relatively resistant to disc degeneration in the earlier years of life [130,131]. Strikingly, in chondrodystrophic breeds, notochord cells in the NP are replaced by small nucleus pulposus cells in the first year of life and IVD degeneration proceeds rapidly thereafter [132]. Conversely, in non-chondrodystrophic dogs notochord cells persist in the IVD throughout life [133,134], and while these breeds experience some degree of disc degeneration, it occurs at a much slower rate.

Mouse models are also commonly used to study human disease due to the ease of their genetic manipulation, highly conserved genome, comparable physiology, short gestation period and low housing costs [135,136]. The murine NP is predominantly composed of notochord cells until skeletal maturity, and like humans, there is a progressive decline in notochord cells and a concomitant increase in nucleus pulposus cells with age [137]. An important tool in mouse genetics is Cre/loxP-based conditional mutagenesis. In this system, expression of Cre recombinase can be regulated by tissue-specific and/or temporally-regulated promoters, leading to excision of loxP-flanked (“floxed”) genes via intra-chromosomal recombination leading to gain or loss of gene expression. This strategy confers the ability to track specific cell types over time, or assess the function of a specific gene within a given cell type based on the specificity of Cre recombinase expression [138].

### 5.2. Notochord Associated Cre Mouse Strains

Several genes or gene loci have been targeted to generate Cre expressing mouse strains that demonstrate Cre expression in the notochord, including the T-box transcription factor brachyury, *T*-Cre [139], flat-top (*Cfap126*), a cilia and flagella associated protein, *Fltp^T2AiCre^* [140], and type-II collagen *Col2a1*-Cre [141,142,143,144]. However, in these strains Cre expression is not notochord-specific, and is detected in the lateral mesoderm, the neural tube floorplate, head mesenchyme and cartilaginous tissues, respectively. Consequently, novel notochord-specific Cre strains were required to trace the developmental lineage of NP cells. The first reported and most commonly used notochord-Cre mouse is the *Shh*-Cre strain [145], which when crossed with a conditional lacZ reporter marked cells throughout the notochord at E10.5, notochord cells during segmentation at E12.5, and the presumptive NP at E16.5 [61]. Importantly, no β-galactosidase-positive cells were detected in the surrounding AF or CEPs. To ensure that detection of Cre expression in the postnatal NP was not associated with the induction of *Shh* expression in mature NP cells, a tamoxifen-inducible *Shh-CreER^T2^* [145] was used to transiently induce Cre expression in the notochord between E8.5–E12.5. This experiment conclusively demonstrated that all the cells the mature NP (in mice ≥19 months of age) were derived from the *Shh* expressing cells of the node and notochord [61].

The sickle tail (*Skt*) gene has been reported to play a role in the development of the notochord and IVD. The Skt protein is localized to the cytoplasm where it is thought to act as a structural element or a scaffolding protein. *Skt* was an interesting target for the development of a notochord-specific Cre strain since in gene-trapping experiments, intense β-galactosidase expression was detected in the notochord and at birth the mice had kinky tails, compressed IVDs, and disorganized NP tissues [146]. *Skt*-Cre mice were subsequently generated and when crossed to a conditional LacZ reporter, β-galactosidase expression was detected in the notochord at E9.5 and E12.5. At E15.5 β-galactosidase expression was predominantly detected in the NP however, small cell populations were also detected in the primitive AF. In adult mice, β-galactosidase expression was detected in the NP but large populations of AF were also LacZ positive [147], making the *Skt*-Cre mouse not suitable for NP-specific genetic experiments.

Forkhead box protein A2 (FoxA2), also known as hepatocyte nuclear factor 3-β, is a transcription factor necessary for the formation and maintenance of the definitive endoderm [148] and required for node and notochord formation [149]. Both a *FoxA2*-Cre strain [150,151] and an inducible *FoxA2^mcm^* Cre strain [150] have been generated, and when crossed to a conditional Rosa26-LacZ reporter mouse, β-galactosidase expression was detected in the endoderm but also in the node, floorplate, and notochord [150]. *FoxA2*-Cre has been used to knockout hypoxia inducing factor-1α, which demonstrated a NP phenotype [152].

Lastly, work form our group targeted the *Noto* locus that encodes a homeobox transcription factor that is transiently but specifically expressed in the node and the posterior notochord between E7.5–E12.5. Noto has been shown to regulate morphogenesis, node ciliogenesis and left-right embryo patterning [153]. Characterization of Cre specificity in this mouse showed specific localization to the node, notochord and NP during development and in adult mice [62], and this mouse strain has subsequently been used to study the role of notochord-derived factors in NP biology [108].

## 6. IVD Disease and Repair: A Mirror of Development?

Basic research and the use of animal models have demonstrated the role of the notochord as a regulator of disc development, enabling investigations into the ability of notochord-associated signals to modulate disc degeneration. One example is the bone morphogenetic protein (BMP) pathway which is critical for embryonic notochord patterning [154], IVD development, and is also reactivated during disc degeneration in rabbits [155] and rodents [156] localized to the NP and CEP [157]. Consequently, BMPs have been evaluated as potential therapeutic targets to treat disc degeneration, owing to their ability to promote ECM gene expression [158] and their anti-apoptoic effects [159]. However, the use of recombinant BMPs to treat disc degeneration has been called into question due their potent ability to induce osteogenesis leading to spinal fusion [160], and associated complications including swelling in the cervical spine [161]. It remains unclear whether the increased BMP signaling detected within the degenerate IVD is a result of the degenerative cascade or an indication of a reparative tissue response; further studies are required to elucidate the role of BMP in disc degeneration.

The Wnt/β-catenin pathway has been shown to maintain notochord progenitor cell fate during early notochord formation and is required for posterior extension of the notochord [162]. β-catenin is also critical in IVD development. Targeted ablation of β-catenin in type II collagen expressing cells lead to CEP calcification; conversely, ectopic activation of β-catenin in the AF and vertebral growth plate resulted in severe deterioration of both structures [163]. This may be a direct effect of Wnt/β-catenin activation or an indirect effect mediated by altered SHH signaling, given the feedback between these two pathways in the NP [164,165]. Interestingly, in the adult NP expression of both SHH and Wnt/β-catenin are downregulated, as are Wnt targets in the whole IVD [164]. The down-regulation of Wnt/β-catenin signaling in the aged disc has been shown to be reversible and that re-activation of this pathway can induce molecular markers of the IVD that was lost in degeneration [164]. Interestingly, canonical Wnt signalling has also been shown to be upregulated in the NP during early disc degeneration in chondrodystophic dogs [166]. Taken together, these findings suggest a dual role for Wnt/β-catenin in the IVD and its potential as a therapeutic intervention for degenerative disc disease.

Brachyury is a highly conserved transcription factor that is expressed in the developing notochord, required during embryogenesis for proper development of the primitive streak, axial and posterior mesoderm [167]. Brachury is expressed in notochord cells of the NP but not in the nucleus pulposus cells of mature rat discs [115]. Interestingly, increased expression of brachyury, resulting from either gene mutation [168] or gene duplication [169], has been associated with the notochord-derived tumour termed chordoma. Chordoma is a rare invasive cancer responsible for 20% of primary spine tumours, located at clavius of the skull (32%) and sacrococcygeal region (29%), and less frequently in cervical, thoracic and lumbar vertebrae [170,171]. Chordomas are morphologically similar to notochord cells, formed by physaliferous cells that co-express notochord cell genes including cytokeratins 8, 18, and 19 and brachyury [79]. Fate mapping of the notochord demonstrated that during disc formation, not all notochord cells are incorporated in the IVD; some persist in the vertebral bone [61,62]. In humans, notochord cell remnants have been detected in 20% of adult vertebrae but do not develop into neoplasms [172]. The current hypothesis is the chordoma formation results from the activation and proliferation of notochord remnants; while the pathways regulating this process have yet to be elucidated; it is intriguing that malignant transformation is associated with the activation of developmental notochord factors such as brachyury.

## 7. Conclusions

The IVD is a highly specialized tissue that requires complex developmental patterning to form a complex joint. The notochord and notochord-derived cells play an integral role in the development of the embryo and continue to be important in maintaining IVD homeostasis. Studies to date suggest that as the IVD undergoes degeneration, reactivation of these pathways may not contribute to tissue breakdown but instead reflect the cellular attempt to activate a program of tissue repair. Future studies are needed to understand these processes and exploit them as potential therapeutics for disc degeneration.
